# Serum Concentrations of Osteogenesis/Osteolysis-Related Factors and Micro-RNA Expression in ST-Elevation Myocardial Infarction

**DOI:** 10.1155/2019/1420717

**Published:** 2019-06-02

**Authors:** Tomasz Jadczyk, Tomasz Francuz, Wojciech Garczorz, Malgorzata Kimsa-Furdzik, Mieczyslaw Dutka, Ewa Gaik, Katarzyna Gruszczynska, Marcin Syzdol, Wojciech Wanha, Radoslaw Kurzelowski, Joanna Fluder, Zdenek Starek, Wojciech Wojakowski

**Affiliations:** ^1^Department of Cardiology and Structural Heart Diseases, Medical University of Silesia, Katowice, Poland; ^2^Interventional Cardiac Electrophysiology Group, International Clinical Research Center, St. Anne's University Hospital Brno, Brno, Czech Republic; ^3^Department of Biochemistry, Medical University of Silesia, Katowice, Poland; ^4^Faculty of Health Sciences, Department of Biochemistry and Molecular Biology, University of Bielsko-Biala, Bielsko-Biala, Poland; ^5^American Heart of Poland, Dabrowa Gornicza, Poland; ^6^Department of Diagnostic Imaging, Medical University of Silesia, Katowice, Poland; ^7^1st Department of Internal Medicine—Cardioangiology, St. Anne's University Hospital Brno, Brno, Czech Republic

## Abstract

**Background:**

Atherosclerosis and bone metabolism share similar molecular and cellular mechanisms. This study aims to evaluate (1) serum concentration of osteogenesis/osteolysis factors panel (Dickkopf-related protein 1 (DKK-1), TNF-*α*, *N*-terminal atrial natriuretic peptide (NT-proANP), thrombospondin-2 (TSP-2), osteoprotegerin (OPG), osteocalcin (OCN), osteopontin (OPN), fibroblast growth factor 23 (FGF-23), soluble receptor activator of nuclear factor-kappaB ligand (sRANKL), tumor necrosis factor-related apoptosis-inducing ligand (TRAIL), proprotein convertase subtilisin/kexin type 9 (PCSK9)), (2) serum expression levels of micro-RNA- (miR-) 24-1 and miR-6802, and (3) assess their correlation with myocardial injury and LV remodeling and function in the acute phase of STEMI and after 3 months.

**Methods:**

Study enrolled 25 STEMI patients (mean age 55.4 ± 8.96 years). Blood samples were collected 4 days and 3 months after myocardial infarction. Serum concentrations of osteogenesis/osteolysis factors were measured using the Luminex assay. Analysis of miR-24-1, and miR-6802 expression was performed with qPCR. LV function and remodeling were assessed by MRI during index hospitalization and 3 months later.

**Results:**

There were no significant differences in serum levels of osteogenesis/osteolysis factors and expression of miR-24-1 and miR-6802 between the acute phase and 3-month follow-up. The levels were similar in patients with at least ≥5% improvement of LVEF (*n* = 10) and those without improvement. There was a negative correlation between the OPG serum level and LVEF during the acute phase of myocardial infarction.

**Conclusions:**

In STEMI patients, serum concentrations of osteogenesis/osteolysis factors, as well as miR-24-1 and miR-6802 expression, do not change significantly within the 3-month follow-up and are not correlated with LV remodeling and function.

## 1. Introduction

Along with population aging, the prevalence of both ischemic heart disease and osteoporosis increases. The calcification process of arteries depends on mechanisms that share similar regulatory pathways as involved in bone metabolism. An inverse correlation between bone mineral density (BMD) and coronary arteries calcification has been already demonstrated [1, 2]. Interestingly, similar mediators of inflammation, growth factors, and chemokines play an essential role in the remodeling processes of both bone tissue and vascular wall [3, 4]. Correspondingly, similarities of bone turnover with formation and rupture of atherosclerotic plaque initiated an intensive research activity on osteogenesis/osteolysis regulators including osteoprotegerin (OPG), osteocalcin (OCN), osteopontin (OPN), fibroblast growth factor 23 (FGF-23), soluble receptor activator of NF-kappaB ligand (sRANKL), tumor necrosis factor-related apoptosis-inducing ligand (TRAIL), Dickkopf-related protein 1 (DKK-1), TNF-*α*, *N*-terminal atrial natriuretic peptide (NT-proANP), thrombospondin-2 (TSP-2), and proprotein convertase subtilisin kexin type 9 (PCSK9). Importantly, not only bone tissue metabolism markers might have useful prediction value in cardiovascular diseases (CVDs) risk stratification. Recently, micro-RNAs (miRs), a group of small, noncoding RNAs, which regulate gene expression at the transcription and posttranscription level, are evaluated as potential clinically applicable biomarkers. It has been shown that a wide range of miRs take part in postmyocardial infarction (MI) left ventricular remodeling (LVR) [5]. However, the data concerning the prognostic value of bone metabolic markers and miRs in CVD risk assessment are modest and often inconsistent and require further investigation.

Thus, the current study aimed at exploring the concentration of osteogenesis/osteolysis factors and selected miRs expression in the myocardial injury induced by ST-segment elevation myocardial infarction (STEMI).

## 2. Materials and Methods

### 2.1. Patients

The study population consisted of 25 STEMI patients (mean age 55.4 ± 8.96 years) treated with percutaneous coronary intervention (PCI) and drug-eluting stent(s) implantation on the infarct-related artery. The study adhered to the principles of the Declaration of Helsinki and was approved by the Ethics Committee of the Medical University of Silesia in Katowice. The project was funded by the National Science Center grant 2016/21/B/NZ5/01902 (WW).

#### 2.1.1. Inclusion Criteria

Inclusion criteria were as follows: (1) age 18–75 years, (2) first-time MI, (3) STEMI diagnosed according to the European Society of Cardiology guidelines and referred for primary PCI within 12 hours after the onset of the chest pain, and (4) signed written informed consent.

#### 2.1.2. Exclusion Criteria

Exclusion criteria were as follows: (1) history of myocardial infarction within 30 days prior to study enrolment, (2) history of coronary artery intervention or CABG within 30 days prior to study enrolment, (3) pregnancy, (4) neoplasm, (5) chronic kidney failure (eGFR <30 ml/min/1.73 m^2^), (6) liver failure, (7) osteoporosis, (8) autoimmunological disorder, (9) chronic treatment with steroids or bisphosphonates, (10) chronic obstructive pulmonary disease, (11) active infection, and (12) bone injury within 30 days prior to study enrolment.

### 2.2. Laboratory Investigations

Blood (5 ml) was collected from each subject 4 days and 3 months post-PCI. Whole blood samples were obtained in Vacutainer tubes (Becton Dickinson and Company, Franklin Lakes, NJ, USA) containing a coagulation activator and were left to clot for 15 minutes. Afterward, the samples were centrifuged, aliquoted, and stored frozen at −176°C until further analysis.

#### 2.2.1. Serum Concentration of Osteogenesis/Osteolysis Factors

Before analysis, with the lids tightly secured, the tubes were placed in a water bath and heated at 15–20°C. After thawing, samples were centrifuged for 15 min at 1000 g (15°C) to remove precipitates if any formed. After that, the supernatant was diluted 1 : 1 by transferring 35 *μ*l of serum into a new tube containing 35 *μ*l of analyzed protein-deprived serum-like buffer. As a quality control, lyophilized human serum was used. Before analysis, lyophilisates and calibration standards were dissolved in distilled water. Sample analysis was performed at 22°C.

Analysis of osteogenesis/osteolysis factor serum concentrations was carried out using commercially available, high-sensitive Luminex kits: (1) DKK-1, TNF-*α*, OPG, OCN, OPN, and FGF-23—EMD Millipore Corporation, and (2) NT-proANP, PCSK9, TRAIL, sRANKL, TSP-2—R&D Systems. The analyses were carried out according to the manufacturer's instructions in duplicates.

#### 2.2.2. Serum Expression of miR

Total RNA, including small RNAs, was extracted using the miRNeasy Serum/Plasma Kit (Qiagen), according to the manufacturer's instructions. In brief, 200 *µ*L of serum was used, and samples were treated with Dnase to remove DNA. To provide an external standard, an appropriate amount of Spike-In Control −5.6 · 10^8^ copies of Caenorhabditis elegans, miR-39-3p was added to each sample after the addition of QIAzol Lysis Reagent.

Reverse transcription was performed with miScript II RT Kit (Qiagen) according to the manufacturer's instructions. Negative controls (without the cDNA template) were similarly processed along with the serum samples. Specific primers for each miRNA were used (Table 1). qPCR analysis was done with miScript SYBR Green PCR Kit (Qiagen) using LightCycler 480 machine (Roche) with 45 amplification cycles. At the end of the amplification program, melting analysis was performed to confirm the presence of a single PCR product. For serum samples, cel-miR-39-3p was used for normalization. Relative quantification was performed using the 2^−ΔCp^ method, where ΔCp = Cp_target_ − Cp_cel-miR-39_.

Routine laboratory analytical methods were used to assess complete blood count and plasma concentration of troponin I, CK-MB, creatinine, glucose, sodium, potassium, total cholesterol, LDL cholesterol, HDL cholesterol, and triglycerides.

### 2.3. Imaging Modalities

#### 2.3.1. Cardiac Magnetic Resonance

In all patients, cardiac magnetic resonance (CMR) studies were performed 4 days and 3 months post-PCI procedure using a 1.5 T unit (Magnetom Avanto, Siemens, magnetic gradient amplitude 40 mT and slew rate 200 mT/m/s, with a 4-element phase-array receiver coil. Examination protocol consisted of localizers and ECG-gated b-SSFP sequences in LV 2-ch, 3-ch, and 4-chamber view and a stack of cine images in LV short axis, 8 mm slices without a gap, from the supravalvular plane to apex (CMR examination parameters used for the b-SSFP sequence are TE 1.2–1.4 ms, FA 67°, number of averages, 19 temporal phases per slice, 24 segments, voxel size 2.1 × 2.1 × 8 mm, and repetition time 3.0–3.6 ms). Parameters of LV function (left ventricle ejection fraction (LVEF), left ventricle end-diastolic volume (LVEDV) and end-systolic volume (LVESV) indexes, stroke volume (SV), cardiac output (CO), and LV mass were calculated on a dedicated workstation (Leonardo, Siemens), using the CMR imaging software (Argus, Siemens). Endo- and epicardial contours were manually drawn on short-axis end-diastolic and end-systolic images. Papillary muscles and endocardial trabeculations were included into LV volume. The correction of the through-plane motion was assessed by the slice-omission technique: basal slices with an incomplete muscular ring of less than 75% were excluded from LV as “atrial.” The infarct area was defined visually as the late-enhancement region on LV short-axis view (multislice FGE IR and phase-sensitive inversion recovery (PSIR) sequence, performed 10 min after injection of gadolinium −0.2 mmol/kg i.v., using, multiple breath holds, trigger on every second heartbeat, diastolic gating, 6 mm slices, adjusted TI for nulling of normal myocardium).

### 2.4. Statistical Analysis

Parametric data were presented as a mean ± standard deviation (SD). Nonparametric data were expressed as a median (interquartile range (IQR)). Qualitative data were reported as crude values and/or percentages. Differences between osteogenesis/osteolysis factors serum concentration and miR expression levels at 4-day and 3-month time-point were analyzed using *T*-test or Mann–Whitney *U* test, in accordance with data distribution. Univariate correlations were examined using Spearman's correlation coefficient test. Data distribution was evaluated with the Shapiro–Wilk test. A value of *p* < 0.05 was considered significant. Data given were analyzed using Statistica 13.0.

## 3. Results

In the majority of cases, the infarct location was inferior and anterior wall. Accordingly, revascularization of the right coronary artery (RCA) and left anterior descending artery (LAD) was performed in most of the cases. Furthermore, single-vessel coronary artery disease (CAD) was diagnosed in 60% of patients. All patients were treated successfully with TIMI 3 flow in the culprit vessel. Representation of CAD risk factors (smoking, hypertension, dyslipidemia, and diabetes) of the study participants reflected a broader population of STEMI patients treated currently in everyday clinical practice. No deaths and adverse events were noted during the follow-up. Tables 2 and 3 present study population baseline characteristics and laboratory findings on admission, respectively.

In comparison to the baseline measurements, there was a trend towards higher OCN serum concentration after 3-month follow-up (*p*=0.06); however, none of osteogenesis/osteolysis factors showed statistically significant change within the duration of the study (Table 4). Correspondingly, based on the CMR results, there was no significant change in LV volumes and LVEF (Table 5). Interestingly, the OPG showed a negative correlation with LVEF during the acute phase of myocardial infarction (*p*=0.01); nonetheless, this observation was not present after 3 months. Moreover, there was no correlation between concentrations of osteogenesis/osteolysis factors and troponin I level; however, despite not significant, improvement of LVEF measured 3 months post-PCI was associated with lower necrotic mass at baseline.

Additionally, as ≥5% change in LVEF is considered clinically relevant, a subgroup of patients with LVEF improvement of at least 5% during the follow-up was identified (*n* = 10). In this study subpopulation, PCSK9 showed trend towards increased serum concentration 3 months after STEMI (Table 6).

Analysis of miR-24-1 and miR-6802 expression levels showed no statistically significant differences when measured 4 days and 3 months post-PCI (Table 7).

## 4. Discussion

From the clinical point of view, it is essential to find a reliable marker of arterial calcification. Based on literature data, reversed correlation between BMD and coronary arteries calcification levels has been reported in the different groups of patients [2, 6]. In a meta-analysis by Ye et al., including more than 10 000 patients, it was showed that decreased BMD is a risk factor for the development of clinically evident arteriosclerosis [7]. Hence, bone metabolism mediators might provide a useful prediction and stratification tool in patients with CAD.

As a next scientific step, we evaluated a potential correlation between LV function and serum concentrations of osteogenesis/osteolysis factors (OPG, OCN, OPN, FGF-23, sRANKL, TRAIL, DKK-1, TNF-*α*, NT-proANP, TSP-2, and PCSK9) in patients with STEMI undergoing the PCI procedure. Additionally, expression levels of miR-24-1 and miR-6802 was included into the analysis.

In the presented study, there were no statistically significant differences in OPG and sRANKL concentrations during AMI and 3 months post-PCI. In contrary, Crisafulli et al. [8] proved the OPG level to increase and sRANKL to decrease in STEMI patients. Furthermore, Halapas et al. [9] demonstrated OPG serum levels to remain elevated even up to 6 months after MI. Moreover, Erkol et al. showed that, in STEMI patients undergoing PCI complicated with a no-reflow phenomenon, OPG serum concentrations were much higher in comparison to individuals with optimal myocardial reperfusion [10]. Thus, in regard to the study results, we presume that the increase of OPG serum level reflects extensiveness of the myocardial necrotic process [11]. In patients with STEMI, OPG levels correlate with a viable myocardium area when evaluated by SPECT [12]. In our study, most probably due to fast reperfusion of the MI-related artery, patients had generally minor myocardial necrosis mirrored by a small ischemic area assessed by CMR and troponin I serum concentrations.

As reported in other studies, there is no direct evidence that sRANKL concentration correlates with myocardial injury [13]. Accordingly, we did not show the increase of this factor (concentrations below detection threshold).

There are at least two TRAIL receptors present in human myocardium (TRAIL-R1 and TRAIL-R2). MI-induced inflammatory response mediates increased expression of TRAIL on CD4+ and CD14+ cells that infiltrate the MI area, which suggests their role in cardiomyocytes apoptosis [14]. However, in our study, there was neither statistically significant change of TRAIL concentrations during the follow-up period nor correlation with LV function parameters analyzed by CMR.

In accordance to previously published studies [15, 16], OCN seems to be a promising biomarker of CAD progression. On the contrary, we did not observe OCN levels to vary in the acute MI phase or at 3-month follow-up. Neither LV function nor MI area was correlated with OCN levels.

Recently, OPN was evaluated as a prognostic factor in patients with stable CAD as well as an indicator for post-MI LV remodeling and coronary arteries calcification [17, 18]. Specifically, in patients with acute myocardial ischemia, increased concentration of this protein was detected in blood samples derived from coronary sinus suggesting OPN release by damaged cardiomyocytes [19]. In our study, we did not confirm the linkage between LV mechanical functions and OPN levels. Moreover, mean levels of OPN measured at the baseline and 3 months after PCI showed no statistically significant difference.

Previously published studies imply FGF-23 as an LV dysfunction predictor [20, 21]. Furthermore, in the Framingham Heart Study serum levels of FGF-23 correlated with total mortality [22], whereas, in LURIC trail, increased FGF-23 was associated with higher mortality in patients with heart failure and reduced ejection fraction [23]. In a presented study, FGF-23 serum levels, similarly to sRANKL, were below the detection threshold of our laboratory equipment. Presumably, only mildly impaired LV function (LVEF around 50%) and effective reperfusion therapy were responsible for this result.

Despite the fact that some authors proved increased levels of DKK-1 during MI [24, 25], we did not observe statistically significant changes in DKK-1 serum concentrations during the follow-up period.

Similarly to DKK-1, no statistically relevant changes of TSP-2 concentrations were noted in our study. No relation between LVEF, including the clinically relevant change defined as ≥5% in comparison to baseline, and LV remodeling was observed. However, some researchers suggest TSP-2 have a better prognostic value in the heart failure group of patients than in CAD [26].

In Ottawa Heart Genomics Study, investigators proved PCSK9 levels were elevated in patients with MI compared to stable CAD [27]. Moreover, Gencer et al. showed that high PCSK9 concentrations correlated with inflammatory mediator levels in patients with MI [28]. However, no difference in PCSK9 serum levels was observed in our study, with no LVEF nor LV remodeling was influenced by PCSK9 concentrations.

Literature data suggests a strong correlation between LV remodeling process and specific miRs expression (e.g., miR-532, miR-145, miR-155, miR-124, miR-1, miR-133a, and miR-208b) [29–31]. Apart from their role in CVD pathophysiology, miRs have been identified in cardiac tissue at all stages of development and are highly expressed in the fetal heart [5, 32]. Based on the large sequencing project, miR-1, miR-16, miR-27b, miR-30d, miR-126, miR-133, miR-143, miR-208, and the let-7 family are highly expressed in nondiseased cardiac tissue, which indicates their role in normal cardiac function as well as the pathophysiology of heart diseases [5, 32]. Explicably, miR-133a shows beneficial effects on infarcted myocardium-stimulating cardiac reprogramming while inhibiting apoptosis and fibrosis. However, miR-133a was also reported to function as an anti-angiogenic factor targeting VEGFR2 and FGFRI. Importantly, recent studies suggest that a balanced level of tissue miR-133a is crucial for the restoration and maintenance of cardiac function. Moreover, circulating miR-133a can be clinically applied as a potential biomarker of MI [33]. This finding is in line with the result previously published by our group, where miR-423-5p plasma level was significantly increased in the acute phase of myocardial ischemic injury [34]. However, miR-24-1 and miR-6802 expression levels did not show similar correlations.

The main limitation of our study is that the number of patients with large infarctions, which could stimulate more significant inflammatory response, was low. Patients enrolled in the study were relatively young, had only mildly impaired LVEF, underwent successful PCI procedure with door-to-balloon time <90 minutes, and preventing unfavorable LV remodeling. Moreover, sRANKL and FGF-23 concentrations were below the detection threshold of our laboratory equipment and thus were not analyzed. Also, it must be noted that osteogenesis/osteolysis and miRs levels were measured in serum, which may not fully reflect tissue concentrations. Regarding study limitations, the presented results require further investigation.

## 5. Conclusion

In STEMI patients, serum concentrations of osteogenesis/osteolysis factors, as well as miR-24-1 and miR-6802 expression, do not change within 3 months of follow-up.

## Figures and Tables

**Table 1:** Description of targets for primers used for qPCR analysis of miRNA.

miRBase accession	Targets mature miRNA
MIMAT0000010	cel-miR-39-3p
MIMAT0027505	hsa-miR-6802-3p
MIMAT0000080	hsa-miR-24-3p

**Table 2:** Baseline characteristics.

Age (mean ± SD)	55.4 ± 8.96
Males, *n* (%)	21 (84)
*MI anatomical localization*	
Anterior wall, *n* (%)	11 (44)
Lateral wall, *n* (%)	13 (52)
Anterolateral wall, *n* (%)	1 (4)
MI complicated with a cardiac arrest, *n* (%)	2 (8)
MI complicated with a cardiogenic shock, *n* (%)	1 (4)

*CAD*	
Single vessel, *n* (%)	15 (60)
Double vessel, *n* (%)	8 (32)
Triple vessel, *n* (%)	2 (8)

*Infarct-related artery with stent(s) implantation*	
Left anterior descending artery, *n* (%)	12 (48)
Right coronary artery, *n* (%)	13 (52)
≥2 vessels, *n* (%)	3 (12)

*CAD risk factors*	
Hypertension, *n* (%)	14 (56)
Dyslipidemia, *n* (%)	5 (20)
Diabetes, *n* (%)	3 (12)
Obesity, *n* (%)	3 (12)
Family history of CAD, *n* (%)	3 (12.5)
Current smoker, *n* (%)	12 (48)

*Pharmacotherapy at discharge*	
Angiotensin-converting enzyme inhibitor, *n* (%)	24 (96)
B-blocker, *n* (%)	23 (92)
Statin, *n* (%)	25 (100)
Aspirin, *n* (%)	25 (100)
Clopidogrel, *n* (%)	25 (100)
Calcium channel blocker *n* (%)	2 (8)
Diuretic, *n* (%)	4 (16)

CAD: coronary artery disease; MI, myocardial infarction.

**Table 3:** Laboratory findings on admission.

Laboratory parameter	Value
Leucocytes (×10^3^/*μ*L)	10.44 (2.72)
Erythrocytes (×10^6^/*μ*L)	4.65 (0.49)
Hematocrit (%)	42.03 (10.47)
Hemoglobin (g/dL)	15.79 (6.70)
Platelet count (×10^3^/*μ*L)	232.60 (51.99)
Troponin I (ng/mL)	0.28 (0.48)
CK-MB (U/L)	64.44 (88.20)
Sodium (mmol/L)	137.32 (3.26)
Potassium (mmol/L)	4.09 (0.47)
Creatinine (mg/dL)	0.83 (0.16)
eGFR (ml/min/1.73 m^2^)	93.70 (11.53)
Glucose (mg/dL)	145 (42.42)
Total cholesterol (mg/dL)	175.30 (27.83)
LDL cholesterol (mg/dL)	110 (23.83)
HDL cholesterol (mg/dL)	38.50 (7.05)
Triglycerides (mg/dL)	133.90 (34.24)

CK-MB, creatine kinase MB isoenzyme; eGFR, estimated glomerular filtration rate. Data are presented as median (IQR).

**Table 4:** Comparison of osteogenesis and osteolysis factors at the baseline and after 3-month follow-up.

Factor (unit)	Baseline concentration	3-month follow-up concentration	*p* value
DKK-1 (pg/ml)	492 (405–630)	430 (358–499)	0.11
TNF-*α* (pg/ml)	1.0 (0.69–1.21)	1.0 (0.82–1.67)	0.29
OPG (pg/ml)	155 (127–182)	155 (137–196)	0.62
OCN (ng/ml)	5.39 (3.88–6.76)	7.20 (4.45–8.76)	0.06
OPN (ng/ml)	17.15 (12.77–22.01)	13.72 (9.68–17.91)	0.10
NT-proANP (ng/ml)	5.52 (4.74–7.66)	5.12 (3.79–8.0)	0.63
PCSK9 (ng/ml)	66.20 (51.77–94.91)	71.49 (54.68–95.47)	0.60
TSP-2 (ng/ml)	25.52 (20.30–40.69)	23.47 (15.78–38.64)	0.39
TRAIL (pg/ml)	62 (61–65)	67 (62–78)	0.10
FGF-23 (pg/ml)	BDL	BDL	
sRANKL (pg/ml)	BDL	BDL	

BDL, below detection level; DKK-1, Dickkopf-related protein 1; FGF-23, fibroblast growth factor 23; NT-proANP, *N*-terminal atrial natriuretic peptide; OPG, osteoprotegerin; OCN, osteocalcin; OPN, osteopontin; PCSK9, proprotein convertase subtilisin/kexin type 9; sRANKL, soluble receptor activator of nuclear factor-kappaB ligand; TNF-*α*, tumor necrosis factor alpha; TRAIL, tumor necrosis factor-related apoptosis-inducing ligand; TSP-2, thrombospondin-2.

**Table 5:** LV function assessed by the CMR at the baseline and after the 3-month follow-up.

Parameter (unit)	Baseline	3-month follow-up	*p* value
LVEF (%)	49.9 (45.9–55.8)	51.6 (43.8–58.1)	0.35
LVEDV (ml)	81.4 (72.2–91.4)	75.3 (67.5–88.0)	0.24
LVESV (ml)	38.5 (33.2–55.6)	36.1 (29.4–47.0)	0.25
SV (ml)	41.5 (37.5–43.9)	39.6 (34.9–43.6)	0.49
CI (L/min)	2.64 (2.1–2.8)	2.3 (2.1–2.8)	0.38
MI mass (g/m^2^)	12.4 (4.2–19.1)	10.3 (3.1–16.4)	0.07

CI, cardiac index; LVEDV, left ventricle end-diastolic volume; LVEF, left ventricle ejection fraction; LVEDV, left ventricle end-systolic volume; MI, myocardial infarction; SV, stroke volume.

**Table 6:** Comparison of osteogenesis/osteolysis factors measured at the baseline between patients with and without ≥5% improvement of LVEF.

Factor (unit)	Patients without at least ≥5% improvement of LVEF (*n* = 15)	Patients with at least ≥5% improvement of LVEF (*n* = 10)	*p* value
DKK-1 (pg/ml)	480 (413–704)	510 (396–555)	0.92
TNF-*α* (pg/ml)	0.91 (0.67–1.22)	1.11 (0.87–1.16)	0.94
OPG (pg/ml)	155 (125–177)	154 (127–209)	0.41
OCN (ng/ml)	5.39 (3.91–6.64)	5.29 (1.34–6.76)	0.82
OPN (ng/ml)	16.12 (14.52–22.57)	19.23 (8.49–22.01)	0.79
NT-proANP (ng/ml)	5.52 (5.02–7.62)	5.82 (3.43–10.71)	0.83
PCSK9 (ng/ml)	60.67 (47.01–83.02)	87.64 (62.76–96.18)	0.07
TSP-2 (ng/ml)	24.93 (20.53–41.19)	26.54 (17.31–32.92)	0.79
TRAIL (pg/ml)	64.4 (61.0–76.7)	61.8 (61.0–62.7)	0.68

DKK-1, Dickkopf-related protein 1; LVEF, left ventricle ejection fraction; NT-proANP, *N*-terminal atrial natriuretic peptide; OPG, osteoprotegerin; OCN, osteocalcin; OPN, osteopontin; PCSK9, proprotein convertase subtilisin/kexin type 9; TNF-*α*, tumor necrosis factor alpha; TRAIL, tumor necrosis factor-related apoptosis-inducing ligand; TSP-2, thrombospondin-2.

**Table 7:** Comparison of miR expression levels in serum samples at the baseline and after 3-month follow-up.

	miRNA level at the baseline (IQR)	miRNA level after a 3-month follow-up (IQR)	*p* value
miR-24-3p	100% (50–250)	83% (17–167)	0.62
miR-6802-3p	100% (50–276)	156% (88–218)	0.42

Relative expressions are normalized to the baseline.

## Data Availability

All data that support the findings of this study are available within the publication.
